# Vehicle‐assisted suicidal strangulation: A case report and literature review

**DOI:** 10.1111/1556-4029.70393

**Published:** 2026-06-19

**Authors:** Marcello Seligardi, Antonio Banchini, Pancrazio Lezzi, Francesco Calabrò, Valentina Bugelli

**Affiliations:** ^1^ Forensic Pathology Section, Medicine and Surgery Department University of Parma Parma Italy

**Keywords:** cervical fracture, forensic autopsy, ligature mark, suicidal strangulation, vehicle‐assisted suicide

## Abstract

Vehicle‐assisted suicidal strangulation is an unusual method of suicide where the victim completes suicide by pulling a ligature tied around their neck using the acceleration of the vehicle they are driving. This report describes a case of a 48‐year‐old man who completed suicide by tying a rope to his neck and to a parked tractor, then using his car to pull the rope. The on‐scene investigation found the body a few meters from his car, which was still running, and a snapped rope tied to a tractor nearby. The autopsy revealed a bruised, encircling ligature mark with underlying injuries to the neck organs and a fracture of the hyoid bone and the C2 vertebra. Additionally, a laceration of the left parietal region was identified. A literature search identified 17 other cases of vehicle‐assisted ligature strangulation, which were compared to the present one to identify common autopsy and on‐scene findings. These cases usually involve high‐intensity forces, which can cause severe wounds atypical for suicidal strangulation and result in messy, puzzling death scenes. As such, this case highlights a rare method of suicide and emphasizes the need for both forensic pathologists and police officers to be aware of this method to avoid misinterpretation of the on‐scene and autopsy findings.

**Highlights**

- Vehicle‐assisted ligature strangulation is a rare and easily misinterpreted suicide method.
- The present case showed a circumferential ligature mark, a deep neck injury, and a C2 fracture.
- Rope rupture and displacement of the body may mimic homicidal strangulation or accidental trauma.
- Correlation of scene findings, autopsy, and literature review is crucial for correct classification.
- This report expands the limited literature on vehicle‐assisted suicidal strangulation.

## 1 INTRODUCTION

Most cases of vehicle‐assisted suicide involve carbon monoxide poisoning or intentional traffic accidents [1, 2]. In other cases, the vehicle is simply used as a safe setting to perform the suicide using methods unrelated to the vehicle itself, such as a gunshot wound, drug overdose, or hanging [3, 4]. It is difficult to assess the exact incidence of vehicle‐assisted suicide, as it is often difficult to distinguish between accidental and voluntary car crashes [5] and in some cases of self‐induced asphyxia [6].

One unusual type of vehicle‐assisted suicide is self‐strangulation, where the ligature is tied to an object outside the car and the acceleration of the vehicle itself is used to tighten the ligature around the decedent's neck. This method of suicide often results in autopsy findings different from those of other cases of self‐strangulation, making a reconstruction of the dynamics of the event more challenging [7]. The authors present one such case of vehicle‐assisted suicide by strangulation, along with a review of the literature on this topic.

## 2 CASE REPORT

**FIGURE 1:**
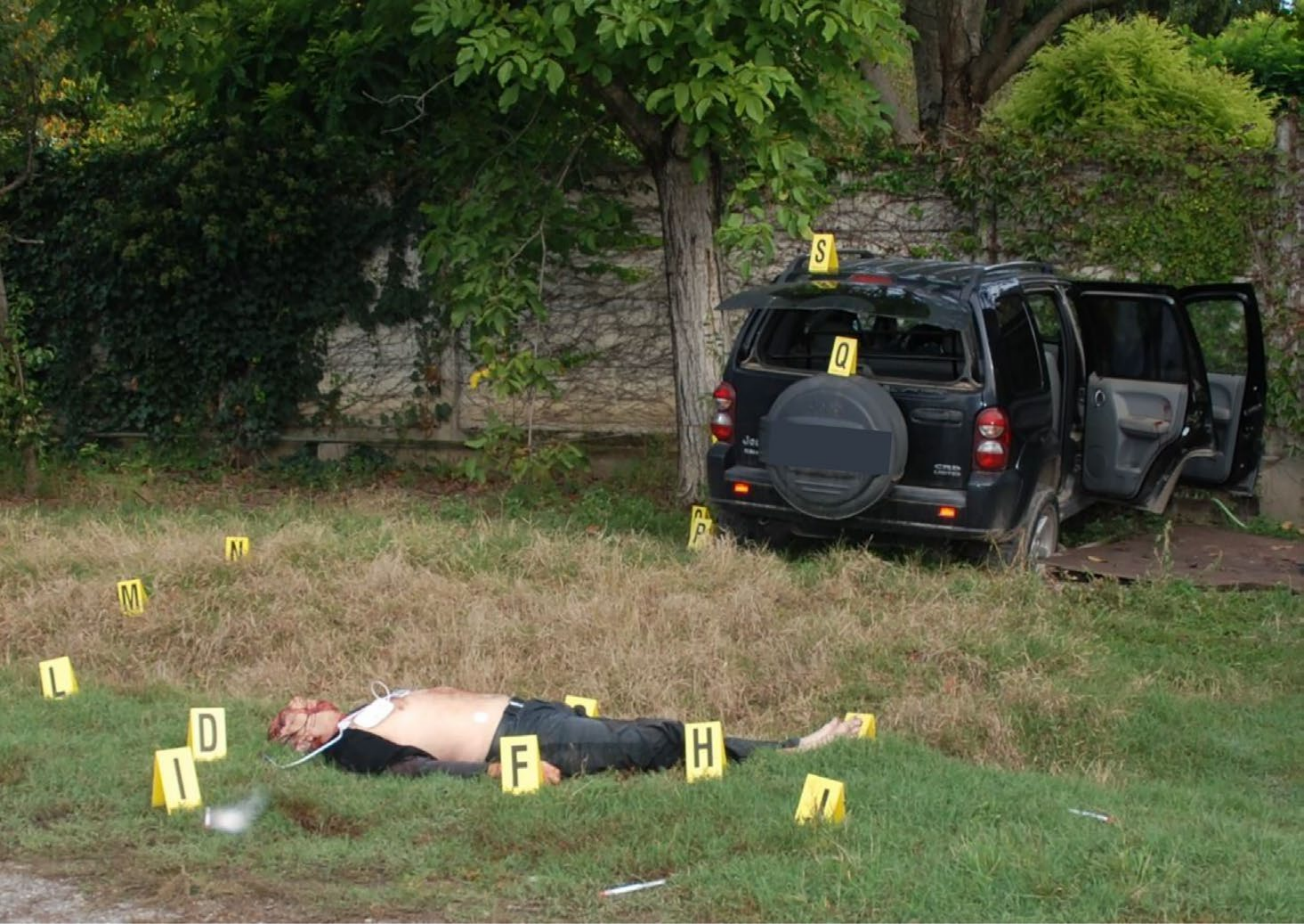
Position of the body and the car. The body was turned face‐up by the emergency responders.

**FIGURE 2:**
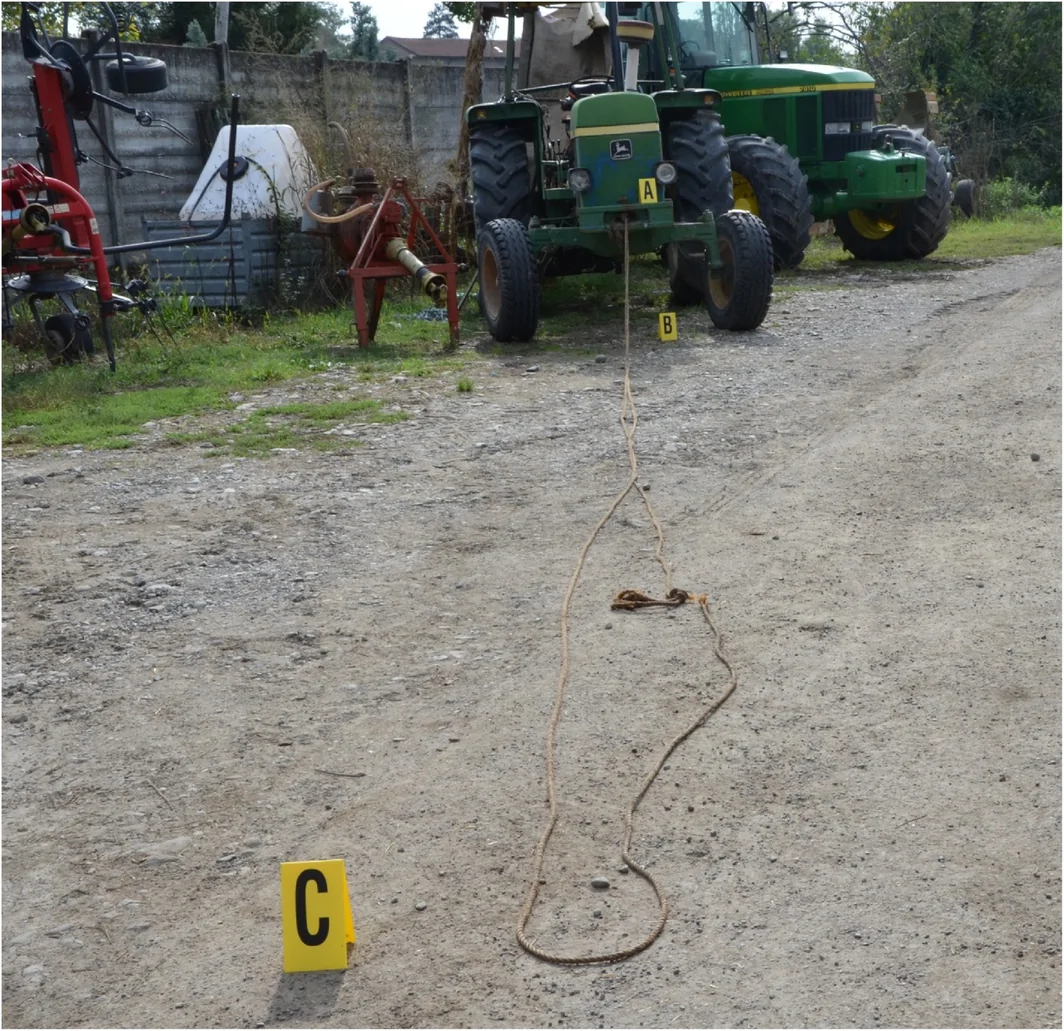
Rope tied to the parked tractor.

In October 2015, the body of a 48‐year‐old white male was found lying face‐down on the side of a dirt road on his farm by one of his employees (Figure 1). His car was found off the road a few meters left of the body, with the engine running and the front pressed against a boundary wall. Inside, the luggage compartment partition grille was damaged and partially removed. Roughly 10 meters to the right of the body, a 1‐cm‐thick rope was found tied to the front of a parked tractor. The rope was snapped near a knot and, when the two halves were put back together, formed a 10‐meter‐long loop (Figure 2). Blood spatter was present on both the front and rear seats of the car, as well as on the path between the car and the body.

**FIGURE 3:**
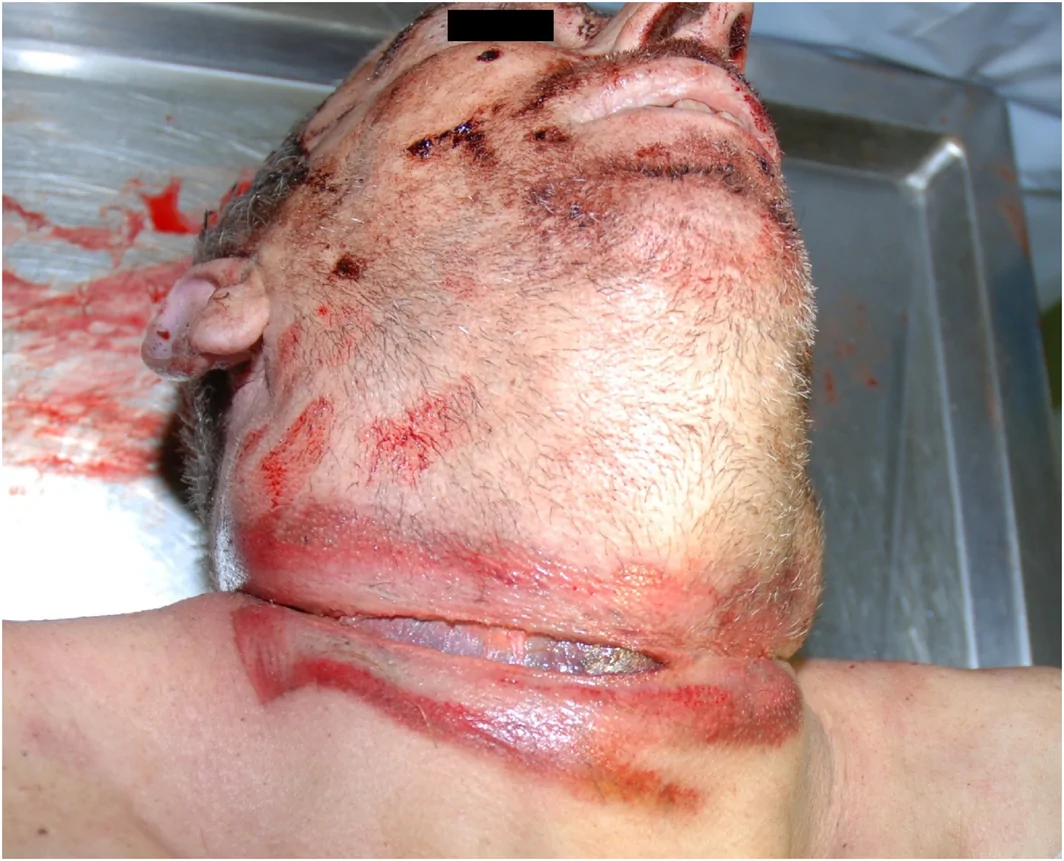
Ligature mark and lacerated wound on the front of the neck.

On external examination of the body, the head and neck were smeared with blood and a 12.5‐cm‐long linear laceration was identified in the left parietal area. A bruised ligature mark ran horizontally below the hyoid bone, completely encircling the neck. On the front and left sides of the neck, inside the ligature mark, was an 8‐cm‐long laceration that ran parallel to the ligature mark itself (Figure 3). Other small bruises and ecchymosis were found on the neck, chest, right scapulae, and left foot.

**FIGURE 4:**
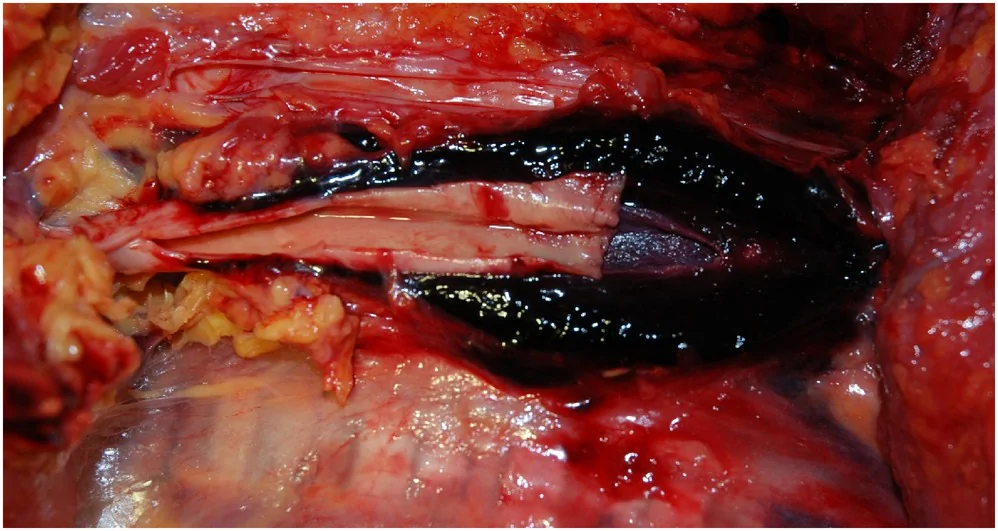
Laceration of the right carotid artery.

The internal examination showed a hemorrhage of the scalp in the parietal region. Examination of the neck showed fractures of both cricoid arches, the left horn of the thyroid cartilage, the left greater horn of the hyoid bone, and the C2 vertebra with a C2–C3 luxation. The right sternocleidomastoid muscle and both carotid arteries were lacerated (Figure 4). All lesions, as well as the thyroid gland and both neck neurovascular bundles, showed hemorrhagic infiltration. The lungs were hyperexpanded with multiple hemorrhagic areas and pleural petechiae. Toxicological exams resulted negative for all substances. A CT scan, performed prior to the autopsy, excluded the presence of gas embolism and confirmed the cartilage and bone fractures found during the dissection.

Police investigation revealed that the farm had recently run into financial troubles, which were worsened by a fire of hay bales a few days earlier.

According to all the findings, a dynamic was recreated in which the man tied an end of the rope to the parked tractor and put a loop on the other end around his neck. He then got into his car and started accelerating until the rope pulled taut and finally snapped, throwing him on the back seats and causing him to hit his head on the partition grille. After this, he was able to get out of the car and walk a few steps before collapsing due to his injuries. As such, the cause of death was classified as ligature strangulation and the manner as suicide.

## 3 MATERIALS AND METHODS

To identify previously reported cases comparable with the present one, in February 2026 we conducted a structured literature search of PubMed, Google Scholar, Scopus, Web of Science, Embase, and the Cochrane Library. The search strategy combined terms related to vehicle‐assisted suicide and ligature strangulation, including “vehicle‐assisted suicide,” “vehicle‐assisted strangulation,” “vehicle hanging,” “car‐assisted strangulation,” “car assisted‐suicide,” “car asphyxia,” and “vehicle‐assisted decapitation.” We included primary case reports or case series describing suicidal ligature strangulation in which a ligature was placed around the neck and tightened by forces generated by a moving motor vehicle, with the opposite end fixed to a stationary object outside the vehicle. We excluded cases of hanging, accidental or autoerotic deaths, and cases without sufficient case‐specific data. Reference lists of all eligible articles were manually screened to identify additional reports. Overall, 16 eligible articles describing 17 previously reported cases were included in the review.

## 4 DISCUSSION

Ligature strangulation is usually seen in a homicidal setting, while cases of suicide are an uncommon event [8]. In these cases, the victim usually sets up a device that will ensure the ligature remains tightly tied around their neck even after he or she has lost consciousness, but there are cases where the unknotted ligature was held in place by the hands of the victim [9]. In cases of ligature strangulation, the evaluation of the manner of death can be extremely difficult and requires a thorough analysis [10]. Evidence that points to a homicide can be the presence of other injuries on the victim (especially defense injuries), the lack of a ligature near the victim, or the inability of the victim to tie the ligature around their neck with enough force (as with children or disabled persons) [11].

Among suicidal strangulation, cases of vehicle‐assisted suicidal strangulation are even rarer, with only a few having been described in literature [12]. These cases are characterized by the fact that the victim uses the acceleration of a vehicle to pull the ligature, which is fixed to an object outside the vehicle itself. A review of the literature using PubMed, Google Scholar, Scopus, Embase, Cochrane, and Web of Science resulted in only seventeen other cases reported in the scientific literature [7, 13, 14, 15, 16, 17, 18, 19, 20, 21, 22, 23, 24, 25, 26, 27], as summarized in Table 1.

**TABLE 1:** Overview of other cases of suicidal strangulation using a vehicle to pull the ligature.

Year	Authors	Location	Age and sex	Body position	Autopsy findings	Toxicology	Ligature (type, integrity, anchor point)
1985	*Hardwicke* et al. [13]	Michigan	67‐year‐old male	Seated in the driver's seat	Face and neck congestion, subconjunctival and pleural petechiae, neck ligature mark, hemorrhages in the neck muscles	Negative	Hemp rope, broken, tied to a forklift
1993	*Prichard* et al. [14]	United States	28‐year‐old male	Seated in the driver's seat	Complete decapitation at the base of the skull	Not specified	Polyethylene rope, intact, tied to a tree stump
2002	*Byard* et al. [15]	Australia	33‐year‐old male	Seated in the driver's seat	Encircling neck ligature mark, bruising of the neck muscles, fractures of the thyroid cartilage, C3, and hyoid bone, transection of right carotid artery, spinal cord, and pharynx	Not specified	Nylon rope, broken at both ends, lying between the vehicle and a wooden post
2002	*Byard* et al. [15]	Australia	24‐year‐old male	Seated in the driver's seat	Horizontal encircling neck abrasion, incomplete decapitation	Not specified	Rope, broken, tied to a post
2004	*Samberkar* [16]	Fiji Islands	63‐year‐old male	Seated in the driver's seat	Face congestion with tongue bite, complete decapitation at the upper third of the larynx	Not specified	Nylon rope, broken, tied to an iron gate
2005	*Türk* et al. [17]	Germany	Male, age not specified	Seated in the driver's seat	Complete decapitation above the larynx, autopsy not performed	Not performed	Metal rope, intact, tied to a fence
2008	*Zhao* et al. [18]	Japan	59‐year‐old male	Seated in the passenger's seat	Face congestion, subconjunctival petechiae, brain edema, complete decapitation between C2 and C3, sternal fracture	Not specified	Hemp rope, intact, tied to a cherry tree
2012	*Morild* et al. [19]	Norway	Male, age not specified	Seated in the driver's seat	Complete decapitation between C3 and C4, fracture of the thyroid cartilage and hyoid bone	Negative	Nylon rope, intact, tied to a lamppost
2012	*Hejna* et al. [20]	Czech Republic	40‐year‐old male	Supine on the driver's and left back seat	Complete decapitation between C2 and C3, head bruises and contusions, blood aspiration	Blood ethanol 0.56 g/L, caffeine, nicotine	Rope, broken, tied to a spruce tree
2013	*Subirana‐Domenech* et al. [7]	Spain	51‐year‐old male	Seated in the passenger's seat with the feet in front of the driver's seat	Discontinuous neck ligature mark, tongue bite, petechiae of the eyelids, conjunctivae, pleura and oral mucosa, surface‐level cuts, hemorrhage and laceration of the neck muscles, thyroid cartilage, esophagus and both carotid arteries, complete tracheal section, brain and lung edema	Not specified	Rope, broken, tied to a bridge
2015	*Madea* et al. [21]	Germany	48‐year‐old male	Supine beside the car	Face congestion, neck ligature mark, petechiae of the conjunctivae and eyelids, hemorrhage of the neck muscles and tongue, fracture of the thyroid cartilage, brain edema	Blood ethanol 0.49 g/L	Rope, broken with an end tied around the neck and the other to a tree
2016	*Akçan* et al. [22]	Turkey	38‐year‐old male	Seated in the driver's seat	Oblique neck ligature mark, thyroid cartilage hemorrhage, bilateral wrist cuts without vessel injury	Negative	Ligature, intact, tied around the neck and to a tree
2018	*Barranco* et al. [23]	Italy	48‐year‐old woman	Seated in the driver's seat	Victim found alive and died shortly after. Subconjunctival and subpleural petechiae, oblique neck ligature mark, laryngeal hemorrhage	Negative	Nylon rope, intact, tied to the neck and wooden fence
2019	*Marchand* et al. [24]	France	43‐year‐old male	Seated in the driver's seat	Face and nail cyanosis, conjunctiva hemorrhage, complete decapitation between C3 and C4, fracture of the thyroid cartilage, lung and brain edema	Not performed	Steel rope, intact, tied to a streetlight
2021	*Ben Amar W* et al. [25]	Tunisia	53‐year‐old male	Seated in the driver's seat	Horizontal discontinuous neck ligature mark, fracture of the thyroid cartilage, edema of the lungs and brain	Negative	Nylon rope, intact, tied to the neck and an electric pole
2023	*Passos* et al. [26]	Portugal	49‐year‐old male	Seated in the driver's seat	Complete decapitation between C1 and C2, fracture of the thyroid cartilage, postmortem head bruises, left wrist cut	Antidepressants and paracetamol	Nylon rope, intact, tied to a wall
2023	*Lorenzoni* et al. [27]	Italy	43‐year‐old male	Seated in the driver's seat	Incomplete decapitation between C3 and C4, encircling oblique neck ligature mark, congestion of the head, hemorrhage of the neck muscles, larynx and aortic root, section of all neck organs and cervical spine, lung edema and intralveolar hemorrhages, brain edema	Blood ethanol 0.57 g/L	Not found
2026	*Present case*	Italy	48‐year‐old male	Prone a few meters from the car	Encircling horizontal neck ligature mark, laceration of the scalp and neck, ecchymosis and laceration of the neck organs, fracture of the cricoid and thyroid cartilage, hyoid bone and C2, pleural petechiae, intralveolar hemorrhage, lung emphysema and edema	Negative	Rope, broken, tied to a tractor

The first case was reported in 1985 by Hardwicke et al. [13]. A 67‐year‐old man was found seated in the driver's seat of his car, with a broken rope nearby tied to a forklift. The internal and external examination showed congestion of the face and neck, subconjunctival and pleural petechiae, and hemorrhages of the neck muscles.

The intensity of the forces involved if the car reaches a high velocity before the rope snaps can result in a decapitation of the victim, as reported by various authors [17, 19, 20, 24, 26]. Zhao et al. [18] described one such case where a 59‐year‐old man tied a hemp rope to a cherry tree and then got in his car, which crashed on the barrier of a bridge after his death. The head of the man was completely severed from the body on a plane passing between C2 and C3. Additionally, the man suffered a sternum fracture and intense brain oedema with tentorial herniation.

In 2015, Madea et al. [21] reported another interesting case. A 48‐year‐old man was found dead, lying supine beside his car. The engine of the car was running, and the driver's door was open. A rope was tied to his neck, corresponding to the ligature mark. While the other end of the rope was broken, the other half was found fixed to a tree 50 meters away from him. The autopsy found massive congestion of the head with subconjunctival petechiae, fracture of the thyroid cartilage, hemorrhage of the left sternocleidomastoid muscle, and brain oedema. The investigators concluded that the man had completed suicide by strangulation using his car but was pulled out of the vehicle through the open driver's door by the force of the rope pulling taut.

In our case, the decedent was a 48‐year‐old male who was found dead outside his car. The demographic aligns with the other cases, where men between 30 and 60 years old are the subjects most often involved. The location of the body, however, is unusual as 16 of the 17 other cases in the literature the body was found inside his car, while in the remaining case, described by Madea et al. [21], the body was found right beside the car, where it had been yanked from the recoil of the rope snapping. In the present case, however, the victim was able to walk a few steps before succumbing to his injuries and was found some meters away from the car.

The type of ligature used in this case, a rope, is similar to those described in many of the other published cases. Likewise, the snapping of the ligature is not an unusual finding, as the forces involved in this method of suicide can easily surpass the resistance of a hemp rope. Interestingly, this can lead to a situation where the ligature is found far from the decedent or cannot be found at the scene at all.

In all cases, the ligature was tied to a stationary object, such as a tree, lamppost, or fence, or to a parked heavy vehicle, such as a tractor in this case or a forklift in the case described by Hardwicke et al. [13] The distance between the anchor point and the body was often large, reaching 100 meters in the case reported by Marchand et al. [24].

Injuries to different body parts different from the neck were described by Zhao et al. [18], and in the present case, respectively a sternum fracture and a left parietal laceration. They can be caused by the car crashing into an obstacle after the owner has lost control of the vehicle, or by the decedent being pulled by the ligature stretching taut and hitting an object.

Another consequence of the intense kinetic energy involved is the severity of the wounds to the neck. Fractures of the hyoid bone, thyroid cartilage, and even cervical vertebrae were described in the present case and multiple others. Neck lacerations, such as the one identified in this case, incomplete or complete decapitation, are also findings common in this type of suicide but virtually absent in other types of suicidal strangulation [28].

In all the cases of decapitation described, the wound is located between C2 and C4 and features a horizontal or oblique direction with perfectly overlapping margins. Additionally, the margins are usually at least slightly bruised and can be either clear‐cut [14, 16] or irregular [18, 26].

All these cases share some common features intermediate between strangulation and blunt force trauma. They all feature a ligature tied around the neck and pulled tight by an external force (the car acceleration) that compresses the vessels and airways, as in strangulation. However, the amount of force applied on the neck often results in much more severe injuries to the soft tissues, cartilages, and even bones, which are more typical of blunt force trauma. In some cases, the mechanism of death is a combination of asphyxia and neck trauma, while in others (as in most cases of complete decapitation), the mechanism is blunt force trauma only. Regardless, the existing literature names many similar cases, even with complete decapitation, as “strangulation” [7, 16, 21, 22, 23, 25, 27].

We've decided to classify the present case as strangulation both to keep uniformity with other articles, and because the mechanism of death is a combination of asphyxia and traumatic injuries, as evidenced by the presence of hyperexpanded, emphysematous lungs with pleural petechiae.

The absence of ligature near the body, the severity of the neck wounds, and presence of other injuries are indicative, in most other circumstances, of homicidal strangulation [11]. As such, care should be taken when evaluating similar cases to avoid misinterpreting a suicide as a homicide or a vehicle accident.

The ligature mark is present in all cases, except when complete or incomplete decapitation occurred, in which case the wound could hide the ligature mark. The mark is described as encircling in five cases, including ours, and discontinuous in two, while in the other four cases its nature is not specified. The direction was horizontal or slightly oblique in all cases. Oher findings typical of asphyxial death, such as face congestion, lung edema, and subpleural and subconjunctival petechia, were found in multiple cases.

Toxicology testing was negative in this case, which is consistent with the other reported cases in which none was found to have any drugs of abuse or blood alcohol concentrations of more than 0.57 g/L. This is in contrast with the literature regarding other manners of suicidal strangulation, where high alcohol concentrations or abused drugs are occasionally found [8]. It is unknown if this difference is only due to the small number of cases reported, or to a real difference in epidemiology.

## 5 CONCLUSION

This case highlights an unusual suicide method using the acceleration produced by a vehicle to pull a ligature around the neck taut. This case, as with similar ones described in the literature, shows severe traumatic lesions and scene findings atypical for a suicide (internal or external decapitation, multiple injuries, body found far from the rope). These findings could render difficult to accurately determine the manner and cause of death, especially if no other clear indicators of suicide are present (for example a written suicide note). A thorough case evaluation, an assessment of all findings, and detailed scene inspection are necessary in order to avoid a misinterpretation of the manner of death in this rare type of suicide.

## CONFLICT OF INTEREST STATEMENT

The authors declare no conflicts of interest.

## Data Availability

Data sharing not applicable to this article as no datasets were generated or analysed during the current study.
